# Treating patients in a safe environment: a cross-sectional study of patient safety attitudes among doctors in the Gaza Strip, Palestine

**DOI:** 10.1186/s12913-020-05230-5

**Published:** 2020-05-07

**Authors:** Maha Alfaqawi, Bettina Böttcher, Yousef Abuowda, Enas Alaloul, Ibrahem Elnajjar, Somaya Elhout, Mysoon Abu-El-Noor, Nasser Abu-El-Noor

**Affiliations:** 1 Nasser Medical Complex, Palestinian Ministry of Health Khan Younis, Gaza Strip, Palestine; 2 Faculty of Medicine, Islamic University of Gaza, PO Box 108, Remal Gaza, Gaza Strip, Palestine; 3 Alshifaa Medical Complex, Palestinian Ministry of Health, Gaza Strip, Palestine; 4 Faculty of Nursing, Islamic University of Gaza, Gaza Strip, Palestine

**Keywords:** Patient safety, Patient safety attitudes, Patient safety training, Doctors, medical errors, Gaza strip, Palestine

## Abstract

**Background:**

Patient safety is important, as in increasingly complex medical systems, the potential for unintended harm to patients also increases. This study assessed the attitudes of doctors in the Gaza Strip towards patient safety and medical error. It also explored variables that impacted their attitudes.

**Methods:**

Doctors, working for at least 6 months in one of the four major government hospitals of the Gaza Strip, were invited to complete a 28-item, self-administered Arabic version of the Attitudes to Patient Safety Questionnaire III (APSQ-III); which assessed patient safety attitudes over nine domains, independent of the workplace.

**Results:**

A total of 150 doctors from four government hospitals participated in this study, representing 43.5% of all 345 doctors working in the four study hospitals at the time of the study. The mean age was 36.6 (±9.7) years. The majority (72.7%) were males, 28.7% worked in surgical, 26.7% in pediatric, 23.3% in medical, 16.7% in obstetrics and gynecology, and 4.7% in other departments. Most participants (62.0%) had never received patient safety training. The overall APSQ score was 3.58 ± 0.3 (of a maximum of 5). The highest score was received by the domain “Working hours as a cause of errors” (4.16) and the lowest score by “Importance of Patient Safety in the Curriculum” (3.25). Older doctors with more professional experience had significantly higher scores than younger doctors (*p* = 0.003), demonstrating more positive attitudes toward patient safety. Furthermore, patient safety attitudes became more positive with increasing years of experience in some domains. However, no significant impact on overall APSQ scores was found by workplace, specialty or whether the participants had received previous training about patient safety.

**Conclusion:**

Doctors in Gaza demonstrated relatively positive patient safety attitudes in areas of “team functioning” and “working hours as a cause for error”, but neutral attitudes in understanding medical error or patient safety training within the curriculum. Patient safety concepts appear to be acquired by doctors via informal learning over time in the job. Inclusion of such concepts into formal postgraduate curricula might improve patient safety attitudes among younger and less experienced doctors, support behaviour change and improve patient outcomes.

## Background

In an increasingly complex medical system with increasing patient demands and choices, the potential for error and unintended harm to patients also increases with potentially serious consequences, including short-term and long-term patient harm or even death [1]. However, certain conditions, behaviours or situations give rise to or prevent patient harm. Among these are the attitudes of staff to patient safety or their ‘safety culture’. Positive patient safety attitudes of staff, where shared working culture recognizes, identifies and establishes systems to reduce risks, as well as learn from error, have been linked to better patient outcomes [2–4]. Conversely, negative attitudes, when potential risks are not recognized or not openly discussed and patient safety is considered to be an individual responsibility, have been linked to poorer patient outcomes [3]. Positive patient safety attitudes can be harnessed by targeted training as well as creating a workplace culture of openness, being aware of potential risks and changing behaviour. This should include an approach to error that is open for learning and development, fostering a non-punitive response to error, which uses errors as an opportunity to learn [5–8]. In order to produce such a work environment for healthcare staff, commitment for patient safety practices and understanding of patient safety concepts must be nurtured on multiple levels, including the organization, the leadership and the workforce. A combination of establishing systems to enhance patient safety practices, learn from errors, improve team functioning combined with patient safety training of leadership and workforce is needed to reach the common goal of safe patient care in complex health systems [2, 4, 5, 7, 9–11]. Therefore, patient safety education has been integrated in postgraduate curricula across the world. However, low- and middle income countries are still lagging behind in this effort [12].

Over the last decades, numerous healthcare interventions have been introduced in an attempt to reduce medical errors and to improve patient safety, but a major barrier has been the organizational culture of healthcare environments [5]. Essential components of safety culture are the attitudes of doctors to medical error, such as disclosure of error, responsibility for error, and it has been suggested that this can be improved by appropriate education in postgraduate training and medical schools [6, 7]. In Palestine, which consists of two geographically separate parts, the West Bank and the Gaza Strip, major efforts have been made in the West Bank to improve patient safety in local hospitals with the patient friendly initiative and partners such as the WHO [13–15]. However, only a limited effect could be shown on patient safety attitudes, even after major investment in this effort with the greatest improvement of only 9.4% having been shown in the area of incidence reporting [13]. Patient safety attitudes of healthcare professionals in the Gaza Strip were found to be moderately positive, when examined in relation to their workplace [16, 17]. Furthermore, nurses showed slightly positive attitudes, when these were examined across different healthcare facilities, with less positive results in areas of recognizing the importance of formal patient safety teaching and the role of individuals in medical error [18]. However, determinants of doctors’ attitudes to patient safety and potential barriers to positive understanding of patient safety within this professional group are not yet well-known.

Therefore, this study aims to assess the attitudes of doctors in the Gaza Strip towards patient safety and medical error, not based on workplace culture and practice, but on individual knowledge and understanding. Furthermore, it explores the impact of gender, age, experience, specialty or previous patient safety training on their attitudes.

## Methods

### Study design and setting

This descriptive, cross-sectional study was performed in four governmental hospitals in the Gaza Strip. There are 13 governmental hospitals located in the Gaza Strip, of which five are larger general hospitals, while the remaining cater for specific patient groups. The selected hospitals for this study were located each in one of the five governorates of the Gaza Strip to achieve a broad representation of healthcare staff. Patients are treated free of charge, when they have the low-cost and universally available government health insurance. All study hospitals offer similar services in the major medical specialties at the same conditions and are representative of the health services available across the Gaza Strip.

### Study population and sample

Eligibility for participation in this study was defined as all doctors, who had worked at least 6 months in one of the four study hospitals. A convenience sample of 154 doctors completed the questionnaire. Of these four questionnaires did not give confirmation of the profession and were therefore excluded from the study. The remaining sample included 150 doctors, representing 43.5% of all 345 doctors working in the four study hospitals during the period.

### Sampling process

Research team members collected the data by approaching doctors, during their work time at one of the four participating government hospitals, asking them to complete a questionnaire. If eligible doctors agreed to participate, they completed the APSQ-III independently in a suitable quiet space and returned it immediately after completion to the research team member. All data were collected and kept anonymously.

### Research instrument

The Attitudes to Patient Safety Questionnaire III (APSQ-III) was the research tool [19]. This had been translated into Arabic and back-translated from Arabic to English by five bilingual researchers with experience in health research from the Faculties of Medicine and Nursing of the local university. The back-translation was done in a separate process to ensure consistency [20]. This translation, was then assessed by five healthcare professionals, working in the government healthcare sector, and adjusted, resulting in a 28-item questionnaire. The resulting Arabic version of the APSQ-III was pilot-tested by 20 healthcare workers with at least 5 years clinical experience [18, 20, 21]. These questionnaires were not included in the current sample. The final version of the instrument showed acceptable reliability with Cronbach’s α of 0.705.

The APSQ-III was developed for use in medical students and covers nine key domains of patient safety attitudes: patient safety training received, error reporting confidence, working hours as an error cause, error inevitability, professional incompetence as an error cause, disclosure responsibility, team functioning, patient involvement to reduce error and importance of patient safety training [19]. This research tool had been judged to be suitable for broader use among healthcare professionals as well as medical students and, thus, was deemed fit to explore doctors’ patient safety attitudes across different workplaces [19].

### Data interpretation

A 5-point Likert scale was used for scoring answers to each individual question, where 1 (strongly disagree) and 2 (disagree) indicated negative patient safety attitudes, 3 (neutral) represented no specific strength of attitude and 4 (agree) as well as 5 (strongly agree) expressed positive responses to the item. Reverse scores were used with eight items of this research tool. These were recoded prior to data analysis [19]. Scores are presented as mean (±standard deviation) for each individual item and for the nine key patient safety domains. Furthermore, the Total APSQ-Scores were calculated by adding the response to all items for each individual participant. These total scores are represented as means (±standard deviations). The Overall APSQ-Score, on the other hand, describing the overall patient safety attitudes as a Likert-scale score with a maximum of 5, was calculated by dividing each individual Total APSQ-Score by the total number of items and then representing this score as one mean (±standard deviation) for all participants. In addition, the proportion of positive responses to each item (defined as a score of 4 and 5) was calculated and is shown as percentage of positive responses per item.

### Data analysis

One-way ANOVA and t-test were used to examine if there were any associations between participants’ characteristics and overall APSQ scores. A *p*-value of ≤0.05 was considered statistically significant. Correlation of the nine domain scores with age and years of experience was tested by Pearson correlation (r) test. Tests were performed with the Statistical Package for the Social Sciences (SPSS) for Windows version 23.

### Ethical considerations

Approval for the present study was obtained from the Human Resources Department of the Palestinian Ministry of Health (MoH), the responsible body for granting approval for studies involving humans. The purpose of the study was fully explained to all participants, all data was collected and kept anonymously and written consent had been taken from all participants prior to completing the questionnaire. Participants were informed that their participation was entirely voluntary and their decision to participate or not had no influence on their employment status.

## Results

A total of 150 doctors from four different hospitals in the Gaza Strip participated in this study, representing 43.5% of all 345 doctors working in the four study hospitals at the time of the study. The mean age of participants was 36.6 (±9.7) years. The majority were males (72.7%), older than 30 years (62%), and took part in residency training (39.0%). Doctors who participated in this study worked mainly in surgical departments (28.7%) followed by pediatric departments (26.7%) with mean years of experience being 9.4 (±8.0). The majority of participants (61.9%) had not received training related to patient safety culture prior to participating in this study (Table 1).

**Table 1:** Characteristics of the respondents and their associations with total APSQ-scores

Variable	Number (%) (***n*** = 150)	Total APSQ score Mean (±SD)	***p***-value
**Gender**			
Males	109 (72.7%)	108.2 (±9.4)	0.031
Females	41 (27.3%)	104.3 (±8.0)	
**Age**			
Mean	36.6 (±9.7)		
≤ 30 years	57 (38.0%)	103.1 ± (9.0)	0.003
> 30 years	93 (62.0%)	109.2 ± (9.7)	
**Years of experience**			
Mean	9.4 (±8.0)		0.005
≤ 5 years	67 (44.7%)	105.4 (±9.3)	
6–10	22 (14.7%)	106.1 (±11.2)	
11–15	24 (16.0%)	108.1 (±8.2)	
> 15 years	37 (24.6%)	110.3 (±8.9)	
**Patient safety training received previously**			
Yes	57 (38.0%)	107.4 (±9.4)	0.793
No	93 (62.0%)	107.0 (±9.1)	
**Professional grade**			
Consultant	31 (20.7%)	109.4 (±8.2)	0.043
Specialist	44 (29.3%)	109.0 (±9.0)	
Trainee/resident	54 (39.0%)	106.9 (±8.4)	
Intern	21 (14.0%)	103.0 (±9.4)	
**Hospital**			
Hospital 1	49 (32.7%)	106.4 (±9.2)	0.231
Hospital 2	80 (53.3%)	107.6 (±9.3)	
Hospital 3	8 (5.3%)	102.2 (±9.0)	
Hospital 4	13 (8.7%)	103.2 (±9.3)	
**Department**			
Medical	35 (23.2%)	106.7 (±8.2)	0.587
Surgical	43 (28.7%)	107.7 (±9.7)	
Pediatrics	40 (26.7%)	105.9 (±9.8)	
Obstetrics and gynecology	7 (4.7%)	106.6 (±9.1)	
Others	25 (16.7%)	109.0 (±8.9)	

The total number of missing values in the 150 questionnaires was 51, representing 1.2% of the 4200, the total number of examined items, which was calculated by multiplying the number of items per questionnaire (28) by the number of participants (150). The missing values were distributed randomly across items, ranging from 0 to 9 missing values per item (0–6.0% per item).

### Attitudes of doctors toward patient safety

The highest score of the nine domains was given to ‘Working hours as a cause of errors’ with a score of 4.2 followed by ‘Team functioning’ with a score of 3.92 (of a maximum score of 5). The domain that received the lowest score was ‘Importance of Patient Safety in the Curriculum’ with a score of 3.25 (Table 2). In total, eight individual questionnaire items had a positive response rate of > 80%, meaning that > 80% of participants scored 4 or 5 on these items. The greatest proportion of participants with positive attitudes was found for ‘Better multi-disciplinary teamwork will reduce medical errors’ (88.7%), followed by ‘Even the most experienced and competent nurses make errors’ (88.0%) and ‘Even the most experienced and competent doctors make errors’ (87.6%; Table 2). On the other hand, less than 40% of participants displayed positive attitudes towards five items, including ‘If people paid more attention at work, medical errors would be avoided’ with only 4.7% giving a positive response, ‘Learning about patient safety issues is not as important as learning other more skill based aspects of being a doctor’ receiving a positive response by only 16.7% of participants and ‘Doctors have a responsibility to disclose errors to patients only if they result in patient harm’ with 28.7%. Interestingly, only 31.3% gave a positive response to ‘I don’t think I make errors’, which means for this reversely scored item that 68.7% of participants think they do not make errors or have no opinion towards this statement (Table 2).

**Table 2:** Results for individual items in means ± standard deviation (SD) as well as percentage of positive responses

	Mean (±SD)	% of positive response
**1. Patient Safety Training Received**		
My training has prepared me to understand the causes of medical errors	3.3 (±1.1)	42.6
**2. Error Reporting Confidence**	**3.3 (±0.7)**	
I would feel comfortable reporting any errors I had made no matter how serious the outcome had been for the patient	3.5 (±1.0)	55.3
I would feel comfortable reporting any errors other people had made, no matter how serious the outcome had been for the patient	3.1 (±0.9)	38.0
I feel confident I could report an error I had made without feeling I would be blamed	3.5 (±1.1)	59.3
I am confident I could talk openly to my supervisor about an error I had made if it had resulted in potential or actual harm to my patient	3.5 (±1.1)	59.3
Medical errors are handled appropriately my workplace	3.0 (±1.0)	35.3
**3. Working hours as a cause of error**	**4.2 (±0.7)**	
The number of hours doctors work increases the likelihood of making medical errors	4.1 (±0.9)	86.0
Shorter shifts will reduce medical errors	4.1 (±0.9)	81.3
By not taking regular breaks during shifts doctors are at an increased risk of making errors	4.3 (±0.9)	85.3
**4. Error inevitability**	**3.7 (±0.6)**	
I don’t think I make errors (R)	2.8 (±1.1)	31.3
Even the most experienced and competent doctors make errors	4.2 (±0.9)	87.3
Even the most experienced and competent make errors	4.1 (±0.8)	88.0
**5. Professional incompetence as a cause of error**	**3.3 (±0.5)**	
Medical errors are a sign of incompetence (R)	3.7 (±0.9)	66.7
Most medical errors result from careless (R)	3.4 (±0.9)	50.7
If people paid more attention at work, medical errors would be avoided (R)	2.1 (±0.8)	4.7
Most medical errors result from careless doctors (R)	3.3 (±1.2)	46.6
**6. Disclosure responsibility**	**3.5 (±0.6)**	
Doctors have a responsibility to disclose errors to patients only if they result in patient harm (R)	2.8 (±1.1)	28.7
All medical errors should be reported	3.9 (±0.9)	70.7
It is not necessary to report errors which do not result in adverse outcomes for the patient (R)	3.4 (±1.1)	47.3
It is the responsibility of all health care professionals to formally report all medical errors which occur	3.7 (±0.9)	64.0
**7. Team functioning**	**3.9 (±0.6)**	
Better multi-disciplinary teamwork will reduce medical errors	4.3 (±0.8)	88.7
Personal input about patient care is well received at my workplace	3.4 (±1.0)	50.7
Teaching teamwork skills will reduce medical errors	4.1 (±0.8)	85.3
**8. Patient involvement in reducing errors**	**3.5 (±0.7)**	
Patients have an important role in preventing medical errors	3.4 (±1.0)	53.3
Encouraging patients to be more involved in their care can help to reduce the risk of medical errors occurring	4.0 (±0.8)	66.7
**9. Importance of patient safety in the curriculum**	**3.2 (±0.6)**	
Patient safety issues cannot be taught and can only be learned by clinical experience when qualified	3.6 (±1.1)	57.3
Learning about patient safety issues before I qualify will enable me to become a more effective doctor	3.8 (±0.1)	74.7
Learning about patient safety issues is not as important as learning other more skill-based aspects of being a doctor (R)	2.3 (±1.1)	16.7

(R) = reversed scored item

### Association between participants’ characteristics and overall APSQ scores

Statistically significant differences were found in the Total APSQ scores in relation to the participants’ age, as older doctors (> 30 years) had higher mean scores than younger ones *p* = 0.001;). Furthermore, male doctors had higher scores than female doctors (*p* = 0.02). However, a larger proportion of male doctors was > 30 years with 82 male doctors (75.2%), compared to only nine female doctors (22.0%). Moreover, the professional grade and years of professional experience showed significant associations with Total APSQ-scores. No further associations were found between the Total APSQ scores with other variables such as specialty, hospital or whether participants had received patient safety training previously or not (Table 2).

Furthermore, correlations between some APSQ-domain scores with the variables ‘age’ and ‘years of experience’, were found (Table 3). Positive correlations existed between age and ‘Patient Safety Training Received’, ‘Error Reporting Confidence’, ‘Professional Incompetence as a Cause of Error’ and ‘Team Functioning’ domains (Table 3). Moreover, a positive correlation existed between years of experience and ‘Patient Safety Training Received’, ‘Error Reporting Confidence’, and ‘Team Functioning’ domains (Table 3). The older the participants and the longer their job experience, the more positive were their attitudes in these domains. No other significant correlations were identified between any other participant characteristics with patient safety attitudes in the examined domains.

**Table 3:** Correlation between age and years of experience and APSQ-dimensions

	Age in years		Professional experience in years	
	r	***p***-value	r	***p***-value
Age in years			0.940	0.000*
Job Experience in years	0.940	0.000*		
D1: Patient Safety Training Received	0.334	0.000*	0.348	0.000*
D2: Error Reporting Confidence	0.267	0.001*	0.229	0.005*
D3: Working hours as a cause of errors	0.019	0.817	0.024	0.769
D4: Error Inevitability	0.164	0.045	0.147	0.072
D5: Professional Incompetence as a Cause of Error	−0.009	0.912	−0.062	0.454
D6: Disclosure Responsibility	0.128	0.118	0.131	0.109
D7: Team Functioning	0.231	0.005*	0.230	0.005*
D8: Patient Involvement in reducing Error	−0.123	0.134	−0.122	0.138
D9: Importance of Patient Safety in the Curriculum	0.098	0.234	0.101	0.219

Significance level: *p* = 0.05; *significant results

## Discussion

This study included 43.5% of the total study population, doctors working at four main governmental hospitals in the Gaza Strip. Overall patient safety attitudes were slightly positive with an Overall APSQ score of 3.56 (±0.3) out of a maximum score of 5, despite the fact that only 38.0% had received previous patient safety training. However, participants displayed relatively more positive attitudes towards the domains ‘Working hours as a cause of error’ and ‘Team functioning’ with 4.2 and 3.9 respectively, compared to almost neutral attitudes towards ‘Importance of patient safety in the curriculum’ with a mean score of 3.2, as well as ‘Professional Incompetence as error cause’ and ‘Error reporting confidence’ with mean scores of 3.3 each. The only variables that showed a positive influence on patient safety attitudes were age, years of job experience and professional grade with increasing age and job experience as well as more senior grades, patient safety attitudes also became more positive. Furthermore, male doctors displayed more positive patient safety attitudes than female doctors, which could be related to the fact that their age and years of experience were substantially higher compared to female doctors. On the other hand, specialty, workplace or whether the doctors had received previous patient safety training had no influence on their patient safety attitudes. Moreover, only three out of the nine domains showed significant correlation to age and professional experience (‘Patient safety training received’, ‘Error reporting confidence’ and Team functioning’ as well as ‘Error inevitability’ also to age).

In concordance with other studies, years of experience significantly impacted patient safety attitudes. Increasing years of professional experience (also reflected again in more senior professional grades in this study) was associated with more positive patient safety attitudes among doctors and nurses in Sweden [22], and among nurses in Pakistan [23], Lebanon [24], Oman [25], Jordan [26], and Palestine [18]. The influence of age follows a similar pattern, probably, as professional experience increases with age. As healthcare professionals gain experience in their profession, they also gain more awareness of risks and will have witnessed adverse events. With this, the awareness of the influence of systems on delivered patient care will grow. Furthermore, with professional experience, confidence also grows and adverse events might be more easily acknowledged by professionals with greater experience, while inexperienced professionals might be hesitant to admit to weaknesses [27]. Okuyama et al. found in a literature review that more experienced professionals are more likely to voice their concerns on patient safety and thus acknowledge patient safety issues more readily [27]. The fact that patient safety attitudes improve with years of experience illustrates a strong degree of informal learning of patient safety issues; globally as well as in the Gaza Strip. However, formal learning of factors improving patient safety has been shown to be more effective, especially when introduced early in medical training [7, 9, 10]. Furthermore, learning by trainees and doctors through witnessing their seniors’ and peers’ actions, has been found to be a barrier to patient safety, due to poor role models or deficient organisational patient safety cultures [7, 28, 29]. Therefore, leadership within hospitals has to demonstrate adoption and support of patient safety concepts in practice. This can be achieved by various interventions, such as providing clear guidelines and procedures, team training in communication or an inviting incidence reporting system with regular staff feedback to support and learn from the process [13–15, 30].

.A reflection of poor patient safety culture in Gaza hospitals might be the limited importance doctors in this study attached to patient safety content in the curriculum, which was the domain with the lowest score. This is surprising, as only 38.0% of participants had actually received patient safety training prior to this study and might be based on poor awareness and little knowledge of this subject rather than actual experience. Patient safety training has been shown to be effective for improvement of patient safety attitudes in various settings, including Palestine [11, 13, 30–35]. Most of these studies monitored self-reported behaviour and attitudes. Only a few studies have, so far, been able to monitor actual changes in behaviour or even patient outcomes, with improvement of patient safety attitudes, such as Hamdan et al., who showed a 9.4% increase in incident reporting following implementation of the WHO patient friendly initiative in Palestinian hospitals in the West Bank [13]. This illustrates that changing staff behaviour and improving outcomes need time and are more difficult to achieve than changes in self-reported attitudes or knowledge. In fact, patient safety training alone can probably not achieve sustainable changes in staff behaviour [31, 36–38]. On the contrary, improvement of patient safety culture within an organsation along with healthcare professional behaviours depends on structural changes within the organisation, positive leadership to achieve such changes as well as improved awareness of crucial patient safety concepts across all staff disciplines and levels [31, 34]. Such changes require commitment from the organisational leadership and established systems that support safe patient care [13, 34–36]. Therefore, it is encouraging that interventions addressing patient safety awareness and attitudes have already shown some positive impact on staff behaviour and patient outcome in different settings, including Palestine [13, 30, 36].

Good team functioning is a key factor in ensuring patient safety. This includes effective communication within and between teams. Teams operate in various contexts in healthcare, on a short-term or a long-term basis [5, 39, 40]. Doctors will be part of different teams throughout their working lives and realizing the importance of effective teamwork, is essential for sustainable patient safety. As previously nurses in Palestine [18], doctors in this study displayed positive attitudes in this domain. In recent years, multidisciplinary team training for the response to emergencies has been delivered and disseminated across Gaza, possibly exerting a positive impact on this domain. However, in this domain, too, organisational culture plays an important role, where all team members are respected, their roles appreciated and they feel comfortable to speak up when necessary [5, 27, 40]. In Gaza, recurring larger scale emergencies, placing heavy strains on healthcare professionals, have given numerous opportunities for doctors to experience the importance of good team functioning. Demonstrating good awareness of the significance of team functioning for patient safety, might be a result of such experiences as well as positive organisational culture in this respect. This is also reflected in other Palestinian studies on patient safety culture, where teamwork is consistently among the highest rated in different settngs [16, 17, 41].

Doctors were found to have almost neutral attitudes in the domains ‘Professional incompetence as a cause of error’ and ‘Error reporting confidence’, demonstrating little understanding of the causes and significance of errors in medicine among doctors. Significantly, although errors were acknowledged to happen to the most competent doctors, they were essentially still regarded as individual failures and little awareness of the role of human factors and systems existed (shown by the negative score on the item ‘If people paid more attention at work, errors could be avoided’). Such weaknesses should be addressed by harnessing an organizational culture across the health system, which values openness, transparency and promotes learning from mistakes. However, the little confidence in reporting of error, illustrates the prevalent culture with punitive responses to errors, instead of using them as opportunities to learn [7, 8, 21]. This difference was also found in a study comparing patient safety cultures in Belgian and Palestinian hospitals, located in the West Bank, where in Palestine a non-punitive response to error was thought to be most important to improve patient safety, while in Belgium, staffing levels were considered more important [41]. The lack of non-punitive responses to errors was identified in two studies in obstetrics, where learning from error and discussing errors freely was hampered by prevalent blame cultures among professionals and families [42, 43]. Such culture remains one of the greatest barriers to learning from error and has been reported by numerous researchers [11, 24, 26, 44–46]. Interestingly, it is a greater problem in low and middle income countries, where delivery of patient safety contents in under- and postgraduate curricula is still poor [12]. Therefore, a profound change of organisational culture is needed to enable professional and organisational learning from mistakes, which can also be achieved in low-resource settings, such as Palestine, including the Gaza Strip [34, 47].

### Strengths and limitations

This study includes 43.5% of the study population with doctors in different hospitals and across specialties and departments, giving a satisfactory representation of patient safety culture at Gaza hospitals. The study instrument used had been designed and validated for use with medical students, but had not yet been validated in the Arabic language version. However, its advantage in the context of this study was that it had been developed to be used independently from workplace culture. This study assessed patient safety culture among doctors from different hospitals and departments, and not in the context of their workplace, therefore, the APSQ-III was thought to be suitable. Furthermore, this study only assessed professionals’ attitudes and not behaviour or outcome. Examination of interventions to improve patient safety attitudes and their impact on healthcare professional behavior are needed to ultimately improve patient outcomes.

## Conclusion

Doctors in the Gaza Strip have slightly positive patient safety attitudes with an overall mean score of 3.56 (±0.3) from a maximum score of 5. However, they demonstrated poor understanding of error in medical practice, as well as poor error reporting confidence, which reflects the prevalent organizational culture in local hospitals, where punitive responses to mistakes prevail. A profound cultural change has to be initiated within the healthcare system; breeding transparent handling of error and enabling individual as well as organizational learning from error. This can be achieved by multi-modal interventions including changes in organizational structures, increased incidence reporting and non-punitive approaches to handling medical error as well as more intensive and consistent patient safety training with content that is relevant to doctors’ professional realities.

## Data Availability

The data generated and analysed in the current study are available from the corresponding author on reasonable request.

## Abbreviations

APSQ-III: Attitudes to Patient Safety Questionnaire, version III
MoH: Palestinian Ministry of Health
SD: Standard deviation
SPSS: Statistical Package for Social Services
WHO: World Health Organization
